# Effect of liver fibrosis on survival in patients with intrahepatic cholangiocarcinoma: a SEER population-based study

**DOI:** 10.18632/oncotarget.27820

**Published:** 2020-11-24

**Authors:** Nimrod Adatto Levy, Guy Kern, Daniel Shepshelovich, Oren Shibolet, Rami Hershkoviz, Ofer Isakov

**Affiliations:** ^1^Internal Medicine “T”, Tel Aviv Sourasky Medical Center and Sackler School of Medicine, Tel Aviv University, Tel Aviv, Israel; ^2^Department of Gastroenterology and Liver Diseases, Tel Aviv Sourasky Medical Center, Tel Aviv, Israel; ^3^Sackler Faculty of Medicine, Tel Aviv University, Tel Aviv, Israel

**Keywords:** liver fibrosis, cholangiocarcinoma, SEER, survival, cirrhosis

## Abstract

Background: Intrahepatic cholangiocarcinoma (iCCA) is a biliary tract malignancy with rising incidence in recent decades. While the causative role of cirrhosis in the development of iCCA is well established, the role of cirrhosis as a prognostic factor in iCCA is debatable.

Materials and Methods: The study population consisted of 512 patients diagnosed with iCCA between 2004–2016 collected from the Surveillance, Epidemiology and End Results (SEER) database. The impact of fibrosis on overall and cancer-specific survival 12, 36 and 60 months following diagnosis, was evaluated in the entire cohort and in sub-groups stratified according to treatment approach and the American Joint Committee on Cancer (AJCC) tumor stage using a Cox proportional-hazards model.

Results: After adjusting for age, sex, race, year of diagnosis, AJCC stage, and surgical treatment strategy, advanced fibrosis was associated with worse cancer-specific survival across follow up periods (HR 1.49 (1.13–1.96, *p* = 0.005); HR 1.44 (1.14–1.83, *p* = 0.002) and HR 1.45 (1.15–1.83, *p* = 0.002) for 12, 36 and 60 months, respectively). Similar effects were observed for overall survival. Among patients that underwent surgical resection, advanced fibrosis was associated with worse overall survival and cancer-specific survival across follow up periods. Fibrosis was associated with worse overall and cancer-specific survival in patients with a later stage (III–IV) at diagnosis but this effect was not demonstrated in early stages.

Conclusions: Patients with iCCA and advanced liver fibrosis have an increased risk of both overall and cancer-specific mortality compared to patients with earlier stages of fibrosis.

## INTRODUCTION

Cholangiocarcinoma (CCA) is a malignant neoplasm of the biliary tract. Accounting for ~15% of all primary liver cancers, it is the most common biliary malignancy [1]. It is usually classified according to its anatomical location - intra-hepatic (within the liver parenchyma; iCCA), and extra-hepatic, which is further divided into perihilar (near the biliary confluence; pCCA) and distal (near the head of the pancreas, along the CBD from the biliary bifurcation to the ampulla; dCCA) [2]. Intra-hepatic disease represents approximately 10% of CCA cases [3]. The incidence of iCCA has increased over the past three decades, both worldwide and in the US, compared with lower incidence of other biliary tract neoplasms [4, 5]. Risk factors for the development of CCA include viral hepatitis, smoking, diabetes and obesity. Specific risk factors identified for iCCA include certain parasitic infections (e.g., liver flukes) and anatomical disorders such as choledochal cysts, hepatolithiasis and primary sclerosing cholangitis [4, 6]. Liver cirrhosis is also associated with CCA [5, 7, 8], especially iCCA [7, 9, 10]. The prognosis of iCCA is unfavorable. Even following curative-intent surgery, the median aggregate overall survival is only approximately 28 months and 5 year survival rarely exceeds 35% [11]. Few prognostic factors have been identified so far. Tumor size, stage and morphology are associated with prognosis and are all included in the American Joint Committee on Cancer (AJCC) staging system. Surgical margins, local lymph node tumor involvement and vascular or perineural invasion markedly affect survival [12, 13]. While there is strong evidence to support the causative role of cirrhosis in the development of iCCA, the role of cirrhosis as a prognostic factor is debatable with contradicting findings from previous studies [14–18].

In this study, we aimed to assess the effect of cirrhosis on outcome in iCCA patients. For this purpose, the largest cohort of iCCA patients to-date was analyzed in order to elucidate the association between advanced liver fibrosis and all-cause mortality and cancer-specific mortality in iCCA patients.

## RESULTS

Out of the entire cohort of patients with iCCA (*n* = 8,390), 905 (10.7%) had information regarding the calculated fibrosis score. In order to validate that patients with an available fibrosis score do not differ from the rest of the cohort, an initial analysis comparing demographic and cancer characteristics was performed (Supplementary Table 1). Patients with an available fibrosis score had a later year of diagnosis (2011 vs 2010), a lower proportion of stage IV disease at diagnosis (32.2% vs 48.9%) and were more likely to undergo any surgical treatment (39.8% vs 21.5%). Other factors including age at diagnosis, sex and race did not differ between groups.

Over the twelve-year period (2004–2016), 512 patients met the inclusion criteria. The study population consisted of 320 patients (62.5%) with a low fibrosis level and 192 (37.5%) patients with advanced fibrosis (Table 1). Compared with patients with low fibrosis levels, patients with advanced fibrosis were more likely to be male (65.6% vs 47.5%; *p* < 0.001) and of white ethnicity (81.8% vs 72.8%; *P* = 0.003). As for cancer characteristics, patients with advanced fibrosis score had a higher proportion of AJCC stage IV (40.6% vs 27.2%; *p* = 0.003). Elevated AFP levels were also more frequent in the high fibrosis score group (42% vs 17.1%; *p* < 0.01). Patients in the advanced fibrosis group were less likely to undergo any surgical procedure compared to those in the low fibrosis group (29.7% vs 45.9%; *p* < 0.001).

**Table 1:** Patient characteristics

		Mild or no fibrosis	Advanced fibrosis	*p*
*N*		320	192	
Age at diagnosis (mean (SD))		63.54 (11.94)	63.21 (9.77)	0.75
Gender (%)	Male	152 (47.5)	126 (65.6)	< 0.001
	Female	168 (52.5)	66 (34.4)	
Ethnicity (%)	White	233 (72.8)	157 (81.8)	0
	Black	21 (6.6)	17 (8.9)	
	Other	66 (20.6)	18 (9.4)	
Year of diagnosis (mean (SD))		2011.24 (3.15)	2011.59 (2.97)	0.21
Tumor size (%)	less than 5	99 (36.1)	67 (42.9)	0.26
	5 to 10	127 (46.4)	69 (44.2)	
	more than 10	48 (17.5)	20 (12.8)	
AFP (%)	Negative/normal	179 (82.9)	83 (58.0)	< 0.001
	Positive/elevated	37 (17.1)	60 (42.0)	
Pathologic grade (%)	well differentiated	23 (18.9)	13 (22.4)	0.72
	moderately differentiated	99 (81.1)	45 (77.6)	
AJCC stage (%)	I	95 (29.7)	57 (29.7)	0
	II	30 (9.4)	17 (8.9)	
	III	108 (33.8)	40 (20.8)	
	IV	87 (27.2)	78 (40.6)	
SEER spread summary (%)	Localized	120 (37.5)	73 (38.0)	0.06
	Regional - direct extension	42 (13.1)	11 (5.7)	
	Regional - lymph nodes	27 (8.4)	13 (6.8)	
	Regional - DEandLN	15 (4.7)	9 (4.7)	
	Distant	116 (36.2)	86 (44.8)	
Surgery (%)	None	173 (54.1)	135 (70.3)	< 0.001
	Liver transplantation	1 (0.3)	9 (4.7)	
	Surgical Resection	137 (42.8)	37 (19.3)	
	Tumor destruction	9 (2.8)	11 (5.7)	
Scope of regional lymph node surgery	Non removed	226 (70.6)	163 (84.9)	0
	Biopsy or aspiration	6 (1.9)	4 (2.1)	
	Number unknown	1 (0.3)	1 (0.5)	
	1–3 lymph nodes removed	48 (15.0)	17 (8.9)	
	> 3 lymph nodes removed	39 (12.2)	7 (3.6)	

**Figure 1:**
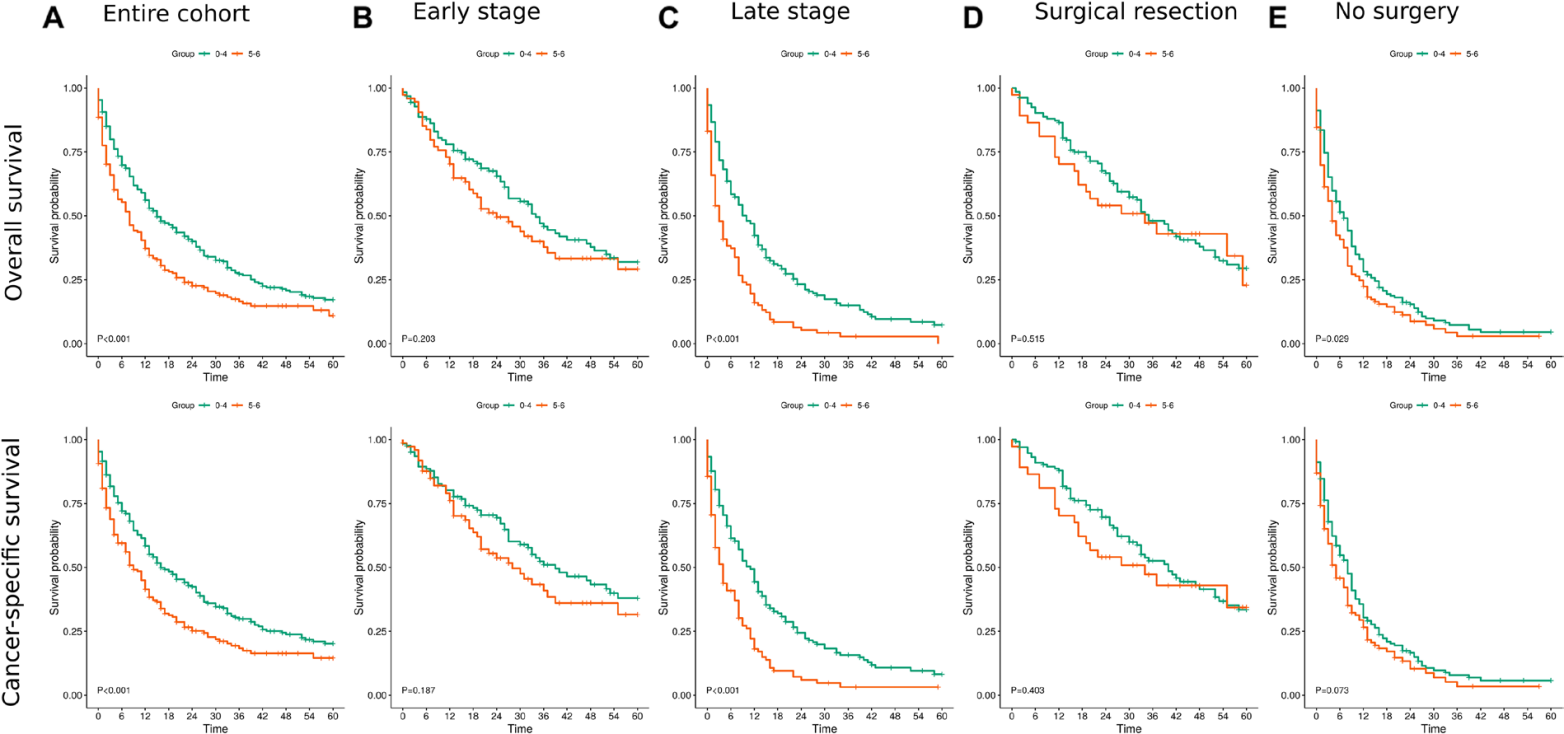
Kaplan Meier survival curves for overall and cancer specific survival. (**A**) For the entire cohort. (**B**) for patients with early stage disease (**C**) for patients with late stage disease (III+IV) (**D**) for patients post surgical resection (I+II) (**E**) for patients who treated conservatively.

Among 512 patients, the incidence of all-cause death at 36 months was 79.2% (152/192) and 66.6% (213/320) in patients with advanced and low fibrosis levels, respectively (unadjusted HR 1.58 (1.28–1.95, *p* < 0.001)) (Figure 1A).

An increased rate of both all-cause and cancer-specific deaths was observed in the advanced fibrosis group across the follow-up periods (12, 36 and 60 months). In a multivariate Cox proportional hazard analysis, adjusted for age, sex, race, year of diagnosis, AJCC stage, and surgical treatment strategy, advanced fibrosis was associated with worse overall and cancer-specific survival across follow-up periods (12M: HR 1.49 (1.15–1.94, *p* = 0.003); 36M: HR 1.43 (1.14–1.80, *p* = 0.002) and 60M: HR 1.44 (1.15–1.80, *p* = 0.001)) and (12M: HR 1.49 (1.13–1.96, *p* = 0.005); 36M: HR 1.44 (1.14–1.83, *p* = 0.002); 60M: HR 1.45 (1.15–1.83, *p* = 0.002)), respectively. Confounders found to be associated with overall and cancer specific survival included older age at diagnosis, earlier year of diagnosis, advanced AJCC stages (III+IV), and certain surgical strategies (surgical resection and tumor destruction) (Table 2).

**Table 2:** Multivariate cox proportional-hazards analysis for 1,3 and 5 Year overall and cancer-specific survival

	Label	levels	HR (1 Yr; *N* = 512)	HR (3 Yr; *N* = 512)	HR (5 Yr; *N* = 512)
Overall Survival	Fibrosis score	0–4	—	—	—
		5–6	1.49 (1.15–1.94, *p* = 0.003)	1.43 (1.14–1.80, *p* = 0.002)	1.44 (1.15–1.80, *p* = 0.001)
	Age (years)		1.03 (1.02–1.04, *p <* 0.001)	1.02 (1.01–1.03, *p <* 0.001)	1.02 (1.01–1.03, *p <* 0.001)
	Sex	Male	—	—	—
		Female	0.99 (0.76–1.28, *p* = 0.925)	0.84 (0.68–1.05, *p* = 0.134)	0.87 (0.70–1.08, *p* = 0.214)
	Race	White	—	—	—
		Black	1.31 (0.80–2.14, *p* = 0.282)	1.28 (0.83–1.98, *p* = 0.261)	1.19 (0.78–1.82, *p* = 0.429)
		Other	0.59 (0.40–0.86, *p* = 0.006)	0.85 (0.64–1.14, *p* = 0.276)	0.84 (0.64–1.11, *p* = 0.228)
	Diagnosis year	Mean (SD)	0.92 (0.88–0.96, *p <* 0.001)	0.93 (0.89–0.96, *p <* 0.001)	0.93 (0.90–0.96, *p <* 0.001)
	AJCC stage	I	—	—	—
		II	1.52 (0.80–2.88, *p* = 0.201)	1.82 (1.16–2.83, *p* = 0.009)	2.05 (1.35–3.11, *p* = 0.001)
		III	2.16 (1.43–3.28, *p <* 0.001)	2.30 (1.69–3.14, *p <* 0.001)	2.38 (1.76–3.21, *p <* 0.001)
		IV	5.20 (3.50–7.73, *p <* 0.001)	4.88 (3.54–6.73, *p <* 0.001)	5.10 (3.72–7.00, *p <* 0.001)
	Surgery	None	—	—	—
		Liver transplantation	0.63 (0.23–1.74, *p* = 0.376)	0.83 (0.38–1.81, *p* = 0.646)	0.85 (0.39–1.84, *p* = 0.677)
		Surgical resection	0.24 (0.16–0.36, *p <* 0.001)	0.32 (0.24–0.43, *p <* 0.001)	0.35 (0.27–0.45, *p <* 0.001)
		Tumor destruction	0.31 (0.11–0.86, *p* = 0.024)	0.26 (0.12–0.56, *p* = 0.001)	0.29 (0.14–0.58, *p <* 0.001)
Cancer-specific survival	Fibrosis score	0-4	—	—	—
		5-6	1.49 (1.13–1.96, *p* = 0.005)	1.44 (1.14–1.83, *p* = 0.002)	1.45 (1.15–1.83, *p* = 0.002)
	Age (years)		1.03 (1.01–1.04, *p <* 0.001)	1.02 (1.01–1.03, *p <* 0.001)	1.02 (1.01–1.03, *p <* 0.001)
	Sex	Male	—	—	—
		Female	1.06 (0.80–1.39, *p* = 0.686)	0.87 (0.69–1.09, *p* = 0.230)	0.91 (0.73–1.14, *p* = 0.409)
	Race	White	—	—	—
		Black	1.33 (0.80–2.20, *p* = 0.269)	1.32 (0.85–2.07, *p* = 0.215)	1.24 (0.80–1.92, *p* = 0.327)
		Other	0.63 (0.42–0.92, *p* = 0.019)	0.90 (0.67–1.20, *p* = 0.463)	0.88 (0.66–1.17, *p* = 0.373)
	Diagnosis year	Mean (SD)	0.92 (0.89–0.96, *p <* 0.001)	0.93 (0.90–0.96, *p <* 0.001)	0.93 (0.90–0.96, *p <* 0.001)
	AJCC stage	I	—	—	—
		II	1.35 (0.66–2.76, *p* = 0.417)	1.65 (1.02–2.68, *p* = 0.042)	1.84 (1.17–2.90, *p* = 0.008)
		III	2.42 (1.55–3.76, *p <* 0.001)	2.51 (1.82–3.46, *p <* 0.001)	2.52 (1.84–3.44, *p <* 0.001)
		IV	5.58 (3.65–8.52, *p <* 0.001)	5.07 (3.62–7.11, *p <* 0.001)	5.20 (3.74–7.24, *p <* 0.001)
	Surgery	None	—	—	—
		Liver transplantation	0.35 (0.09–1.44, *p* = 0.147)	0.64 (0.26–1.58, *p* = 0.334)	0.65 (0.26–1.60, *p* = 0.344)
		Surgical Resection	0.24 (0.16–0.38, *p <* 0.001)	0.32 (0.24–0.43, *p <* 0.001)	0.35 (0.26–0.46, *p <* 0.001)
		Tumor destruction	0.36 (0.13–1.01, *p* = 0.052)	0.28 (0.13–0.62, *p* = 0.002)	0.29 (0.14–0.60, *p* = 0.001)

In a secondary analysis, patients were stratified according to AJCC stage as early (Stage I and II) and late (Stage III and IV). Among patients with early stage, the incidence of all-cause death at 36 months was 56.8% (42/74) and 46.4% (58/125) in patients with advanced and low fibrosis levels, respectively (unadjusted HR 1.35 (0.91–2.01, *p* = 0.136)). The incidence among patients with late stage was 93.2% (110/118) and 79.5% (155/195) in patients with advanced and low fibrosis, respectively (unadjusted HR 2.06 (1.61–2.64, *p* < 0.001)) (Figure 1B and 1C). In a multivariable regression analysis, advanced fibrosis was associated with worse overall and cancer-specific survival across follow-up periods in patients with a later AJCC stage at diagnosis but it was not found to be associated with survival in patients with an early AJCC stage (Table 3).

**Table 3:** Association of fibrosis with 1,3 and 5-year overall and cancer-specific survival by TNM stage

		HR (1 Yr)	HR (3 Yr)	HR (5 Yr)
TNM stage I+II (*N* = 199)	Overall survival	1.03 (0.55–1.91, *p* = 0.926)	1.05 (0.66–1.67, *p* = 0.845)	0.98 (0.63–1.53, *p* = 0.927)
	Cancer-specific survival	0.98 (0.50–1.91, *p* = 0.951)	1.07 (0.65–1.74, *p* = 0.798)	1.07 (0.67–1.71, *p* = 0.790)
TNM stage III+IV (*N* = 313)	Overall survival	1.79 (1.34–2.38, *p <* 0.001)	1.70 (1.30–2.20, *p <* 0.001)	1.72 (1.32–2.23, *p <* 0.001)
	Cancer-specific survival	1.79 (1.33–2.42, *p <* 0.001)	1.69 (1.29–2.22, *p <* 0.001)	1.69 (1.29–2.22, *p <* 0.001)

Among patients that underwent surgical resection (wedge/segmental resection, lobectomy, hepatectomy), the incidence of all-cause death at 36 months was 51.4% (19/37) and 44% (59/134) in patients with advanced and low fibrosis levels, respectively (unadjusted HR 1.21 (0.72–2.03, *p* = 0.473)). In patients that did not receive any surgical treatment the incidence of all-cause death at 36 months was 90% (117/130) and 88.2% (150/170) in patients with advanced and low fibrosis levels, respectively (unadjusted HR 1.32 (1.04–1.69, *p* = 0.023)) (Figure 1D and 1E). In a multivariable regression analysis, advanced fibrosis was associated with worse overall and cancer-specific survival across follow up periods in both patients that underwent surgical resection and in those did not undergo any surgical treatment. (Table 4).

**Table 4:** Association of fibrosis with 1,3 and 5 year overall and cancer-specific mortality by surgical treatment strategy

		HR (1 Yr)	HR (3 Yr)	HR (5 Yr)
No surgery (*N* = 300)	Overall survival	1.39 (1.05–1.85, *p* = 0.023)	1.41 (1.09–1.84, *p* = 0.010)	1.41 (1.08–1.83, *p* = 0.011)
	Cancer-specific survival	1.35 (1.00–1.82, *p* = 0.049)	1.38 (1.05–1.82, *p* = 0.022)	1.37 (1.04–1.80, *p* = 0.025)
Surgical resection (*N* = 171)	Overall survival	4.71 (1.87–11.91, *p* = 0.001)	2.01 (1.09–3.72, *p* = 0.025)	1.85 (1.06–3.24, *p* = 0.031)
	Cancer-specific survival	6.34 (2.33–17.24, *p* < 0.001)	2.30 (1.22–4.34, *p* = 0.010)	2.01 (1.12–3.60, *p* = 0.020)

In a multivariable analysis, with additional possible prognostic factors including tumor size and pathologic grade (*N* = 157 after excluding patients with missing information), advanced fibrosis was the only factor significantly associated with worse overall and cancer-specific survival across all follow up periods (Table 5).

**Table 5:** Association of possible prognostic factors with 1,3 and 5 year overall and cancer-specific mortality

		levels	HR (1 Yr)	HR (3 Yr)	HR (5 Yr)
Overall survival	Fibrosis score	0–4	—	—	—
		5–6	5.56 (2.40–12.89, *p* < 0.001)	2.10 (1.20–3.70, *p* = 0.010)	2.09 (1.23–3.54, *p* = 0.006)
	Tumor size (cm)	< 5	—	—	—
		5–10	1.00 (0.43–2.31, *p* = 0.991)	0.98 (0.56–1.72, *p* = 0.945)	0.99 (0.59–1.64, *p* = 0.958)
		> 10	0.90 (0.30–2.71, *p* = 0.857)	1.34 (0.62–2.87, *p* = 0.455)	1.29 (0.62–2.65, *p* = 0.494)
	Pathologic grade	Well differentiated	—	—	—
		Moderately differentiated	0.64 (0.25–1.61, *p* = 0.343)	0.69 (0.39–1.25, *p* = 0.226)	0.74 (0.42–1.30, *p* = 0.294)
Cancer-specific survival	Fibrosis score	0–4	—	—	—
		5–6	7.80 (3.19–19.06, *p* < 0.001)	2.55 (1.42–4.58, *p* = 0.002)	2.42 (1.39–4.19, *p* = 0.002)
	Tumor size (cm)	< 5	—	-	—
		5–10	1.09 (0.45–2.66, *p* = 0.853)	0.98 (0.54–1.75, *p* = 0.937)	0.96 (0.56–1.64, *p* = 0.885)
		> 10	1.04 (0.34–3.22, *p* = 0.944)	1.42 (0.65–3.09, *p* = 0.376)	1.40 (0.67–2.92, *p* = 0.370)
	Pathologic grade	Well differentiated	—	—	—
		Moderately differentiated	0.61 (0.23–1.59, *p* = 0.310)	0.66 (0.36–1.21, *p* = 0.182)	0.70 (0.39–1.26, *p* = 0.235)

## DISCUSSION

Our study represents the largest cohort to date exploring the association between advanced liver fibrosis (Ishak score between 5 (incomplete cirrhosis) to 6 (cirrhosis)) and survival in iCCA patients. After adjusting for age, sex, race, diagnosis year, AJCC stage and surgical intervention, advanced fibrosis was associated with a 49%, 44% and 45% higher risk of cancer-specific mortality at 12, 36 and 60 months, respectively. An increased risk was also demonstrated for all-cause mortality (49%, 43% and 44% for 12, 36 and 60 months, respectively). In patients undergoing surgical resection, patients with advanced fibrosis were 6 times more likely to die from cancer-specific causes when compared to patients with mild fibrosis during the first 12 months and approximately twice more likely during the first 36 and 60 months. Stratifying the patients according to the AJCC stage, the association between fibrosis and mortality was demonstrated only for patients with advanced stages (AJCC stage III+IV).

iCCA is an aggressive malignant disease with a steadily increasing incidence worldwide. Therefore, the elucidation of factors that may alter the prognosis of this disease has become the focus of interest in multiple recent studies. The association between liver cirrhosis, the most advanced stage of liver fibrosis, and iCCA has been extensively studied. While the presence of cirrhosis has been shown to confer an increased risk for the development of iCCA, its role as a prognostic factor has been the focus of debate. Li et al. [14] examined 113 patients with iCCA treated surgically, among them 32 patients (28%) with cirrhosis, and were the first to conclude that cirrhosis is an adverse prognostic factor. This trend was consistent even in a subgroup analysis of patients with clear surgical margins (R0), in which cirrhotic patients still fared worse than non-cirrhotic ones. On the other hand, other studies assessing prognostic factors of iCCA did not identify cirrhosis as a significant prognostic factor. Endo et al. [15] explored determinants of outcome after surgical resection in 82 surgically treated iCCA patients and found that cirrhosis was not a significant prognostic factor, though only 4 (5%) patients had cirrhosis. Ni et al. [16] assessed 319 iCCA patients who underwent radical resection, among them 123 (38.6%) with liver cirrhosis, and showed that liver cirrhosis had no impact on disease free survival and overall survival. Jeong et al. [17] compared 106 iCCA patients following hepatic resection, 25 (23.6%) of whom had cirrhosis, and found it to be a non significant prognostic factor. Jesper et al. investigated the effects of liver cirrhosis and patient condition on clinical outcomes in 156 patients with iCCA, among them 47 (30%) cirrhotic patients. Their results showed no statistically significant difference in survival between groups regardless of surgical resection or chemotherapy. This discordance between studies could be explained by insufficient cohort sizes and the heterogeneity of patients within the cohorts. In our study we were able to establish the association between advanced fibrosis and survival by utilizing the largest cohort to-date, including 512 patients, 192 (37.5%) of whom with advanced fibrosis, and by stratifying the cohort according to both stage and surgical treatment while characterizing how the association differs between groups. Recently, Zhang et al. [19] analyzed an iCCA cohort derived from the SEER database and found that advanced fibrosis was associated with worse overall and cancer specific survival, and worse overall survival in patients following surgery but not for patients that did not undergo surgery. In their study, no stratification according to tumor stage was performed and a multivariable analysis was used only for overall survival assessment on the entire cohort and not for cancer-specific survival or any of the sub-groups.

Following surgery, the 5-year survival rate for iCCA patients was previously shown to be between 25% to 35%, compared to less than 10% in unresectable disease [20, 21]. Survival of iCCA patients following surgery is influenced by various factors including: tumor size, multifocality, vascular invasion, and lymph node metastases [22, 23]. The prognostic significance of cirrhosis in iCCA patients following surgical resection has been the subject of extensive debate. Patients with cirrhosis were shown to have a high rate of perioperative morbidity and mortality [24–26]. The adverse surgical outcomes in these patients are explained by the high rate of complications, including uncontrolled hemorrhage due to coagulopathy, higher rate of infections, renal failure, hypoalbuminemia and worsening of liver failure [12, 27]. In our study, advanced fibrosis was associated with worse one year overall survival among patients who underwent surgical resection (wedge/segmental resection, lobectomy, hepatectomy), likely due to the increased rate of perioperative complications. Advanced fibrosis was associated with worse cancer-specific survival across follow up periods. This finding might be explained by the fact that surgery in these patients is more complex and results in suboptimal outcomes and therefore reduces the effectiveness of surgical resection in prolonging disease-free survival.

Stratification of iCCA patients into early (stage I–II) and late (III–IV) AJCC stages, demonstrated worse prognosis for patients with advanced fibrosis compared to low fibrosis only at late AJCC stages, with an approximately 70% increased risk of overall and cancer-specific mortality. This difference in association between fibrosis and mortality in early versus late AJCC stages may be explained by different management strategies for each stage. While surgery is the mainstay of treatment in early stage iCCA, it is not recommended in more advanced stages and the use of chemotherapy and local radiation is utilized more frequently. It is possible that the strong association between fibrosis and survival in the advanced stages is conveyed through its effect on the selection of, and response to non-surgical therapy. Our results suggest that, assuming no other contra-indications are present, patients with early stage iCCA and advanced fibrosis are as likely to benefit from surgical resection as patients without fibrosis, stressing the need for additional studies in this sub-group of patients.

In order to explore whether the association between fibrosis and survival is mediated by other known prognostic factors we performed a multivariable analysis including both tumor size and pathologic grade. While tumor size is part of the most recent AJCC staging system, it’s effect on survival has been difficult to establish, with large disparity between studies [14, 28, 29]. In a meta analysis from 2014, including 2132 patients, tumor size was shown to be associated with reduced long-term survival (HR 1.09 (1.02–1.16) for each 1-cm increment) [11]. The prognostic significance of pathologic grade is also debatable. While poor differentiation level was previously shown to be associated with shorter overall survival in cholangiocarcinoma patients, its association with survival in iCCA patients is variable [30–32]. In our study, patients with collected information regarding fibrosis score, tumor size and pathologic grade were analyzed, and advanced fibrosis was the only factor associated with both overall and cancer-specific survival across follow up periods. This further supports our finding that fibrosis is an independent prognostic factor in patients with iCCA.

In this study, in addition to demonstrating worse overall survival in patients with advanced fibrosis, we were able to explore the effect of fibrosis on cancer-specific survival. Among patients who did not undergo any surgical treatment, a multivariable regression analysis showed decreased overall and cancer-specific survival across all follow-up periods. While our results support the known detrimental effects of fibrosis on overall survival, they also suggest that fibrosis results in a more aggressive and malignant tumor behavior. There is accumulating evidence that liver fibrosis is mainly regulated by activated hepatic stellate cells (HSC). This activation of HSCs switches their role from vitamin A-storing pericyte-like cells to α-Smooth muscle actin positive, collagen-producing myofibroblasts. These activated HSC produce numerous angiogenic factors such as VEGF, PDGF, TGF-β which promote tumor vascularization and growth and are actively involved in vascular remodeling [33, 34]. They also release other tumor-promoting cytokines that stimulate tumor vascularization and reduceimmunosurveillance [35]. These microenvironment changes, characteristic of liver fibrosis, may promote tumor progression, and may explain the observed effect of advanced fibrosis on cancer-specific survival.

Our study has several limitations. First, it is a retrospective study prone to weaknesses stemming from this design. Second, although the SEER database includes information regarding whether or not chemotherapy and radiation therapy were performed, this data is incomplete and there is no information regarding which drugs were given, the dose and the duration and therefore this information was not included in our analysis. Third, only a small portion (10.7%) of the entire cohort of patients with iCCA had information regarding the calculated fibrosis score, increasing the risk for selection bias. We addressed this limitation by comparing demographic and cancer characteristics between iCCA patients with a calculated fibrosis score and those without. Our study also has inherent strengths. The large size of the iCCA cohort enabled the stratification of the cohort into specific subgroups according to surgical strategy and tumor stage. Additionally, we were able to demonstrate the association of fibrosis with cancer-specific survival rates in iCCA patients and not only overall survival.

In summary, we show that patients with iCCA and advanced liver fibrosis have an increased risk of both overall and cancer-specific mortality across the follow up period. This association remains significant regardless of whether or not surgical resection was performed. In a sub-cohort of iCCA patients with early stages of the disease, advanced fibrosis was not associated with mortality and therefore, when no other contra-indications are present, should not affect surgical treatment strategy.

## MATERIALS AND METHODS

### Data source

Data for this study was retrieved from the Surveillance, Epidemiology, and End Results (SEER) Program (http://www.seer.cancer.gov/) Research Data (1975–2016), National Cancer Institute, Division of Cancer Control and Population Sciences (DCCPS), Surveillance Research Program, released April 2019, based on the November 2018 submission.

### Study population and data extraction

**Figure 2:**
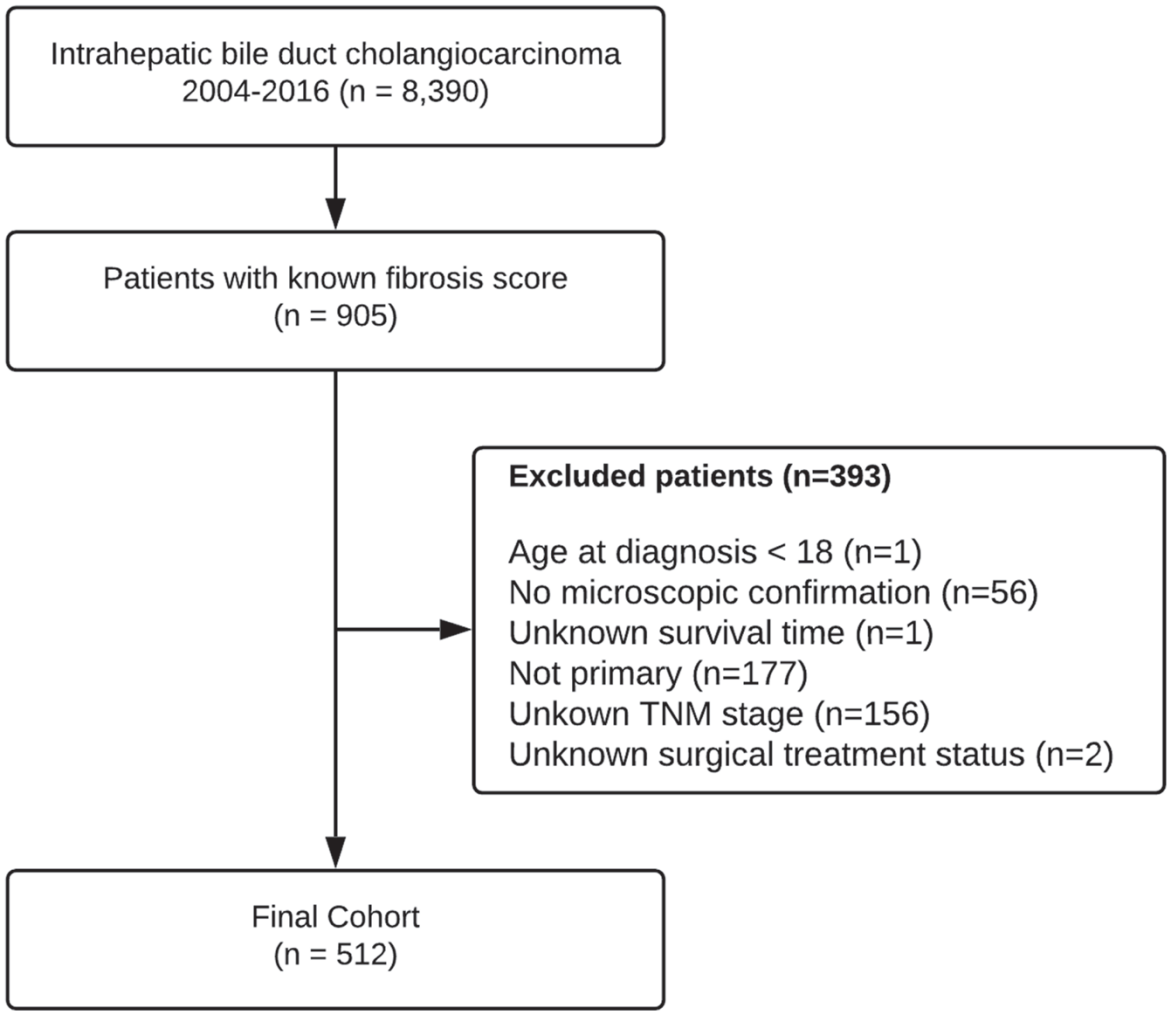
Flowchart depicting patient selection process from SEER database.

The study cohort consisted of all patients with pathologically diagnosed intra-hepatic bile duct cancer (Code C22.1 8160/3 in the International Classification of Diseases for Oncology, third edition, ICD-O-3), between 2004 and 2016. Only patients with a calculated fibrosis score were included. The fibrosis score is divided into two categories, according to the Ishak scale [36]: Low grade fibrosis [score between 0 (no fibrosis) to 4 (fibrosis expansion of portal areas with marked bridging)], and advanced fibrosis [score between 5 (incomplete cirrhosis) to 6 (cirrhosis)]. Patients who were younger than 18 years at diagnosis, had unknown survival time, unknown surgical treatment status, unknown AJCC staging or without microscopic confirmation of the diagnosis were excluded. Only cases in which cholangiocarcinoma was the primary neoplasm or the first cancer of multiple primary cancers were included (Figure 2).

Demographic information regarding each patient’s age, sex, race and clinical characteristics including year of diagnosis, tumor size, AJCC stage, SEER spread summary, alpha fetoprotein (AFP) levels (considered elevated if > 15 ng/ml), fibrosis score and pathological grade were extracted from the database. The SEER spread summary variable includes three categories: (i) “Localized” – no spread beyond the organ of origin or infiltration past the basement membrane of epithelium into stroma of the organ. (ii) “Regional” – extension beyond the limits of the organ of origin, whether direct extension or to regional lymph nodes. (iii) “Distant” – spread to areas of the body distant or remote from the primary tumor. The AJCC stage was based on the Cancer Staging Manual (6th edition) [37]. Surgical treatment was categorized into “None” (if there was no surgical treatment), “Surgical resection” (including: wedge/segmental resection, lobectomy, hepatectomy, “Tumor destruction” (including: photodynamic therapy (PDT), electrocautery; fulguration, cryosurgery, laser, alcohol, heat-radio-frequency ablation (RFA), ultrasound and acetic acid) and “Liver transplantation”). Scope of regional lymph node surgery was categorized into no regional lymph nodes removed, 1–3 nodes removed and 4 or more nodes removed.

### Statistical analyses

Continuous and categorical variables were compared using the Student’s *t*-test and Chi-squared test, respectively. Overall survival was calculated from the date of disease detection until death from any cause. Cancer-specific survival was calculated from the date of disease detection until death associated with the CCA. For the cancer-specific survival analysis, deaths attributed to CCA were treated as events and deaths from other causes were treated as censored observations. Time was censored at the earliest of: one, three and five years after disease detection, the date of last follow-up assessment or, in the cancer-specific group, at the date of death from any cause other than CCA. Survival was estimated using Kaplan–Meier survival curves and compared using the log-rank test. In the primary analysis, hazard ratios (HRs) and corresponding 95% confidence intervals (CIs) for the association between fibrosis and the one, three and five year survival were estimated using a Cox proportional-hazards model. The model included the following potential confounders: age, sex, race, year of diagnosis, AJCC and surgical treatment. Following the primary analysis, three secondary analyses were performed: first, the association between the presence of fibrosis at different tumor stages and survival was estimated by stratifying patients according to the AJCC stage into early (I and II) and late (III and IV) stages. Second, in order to better assess the prognostic significance of fibrosis in patients undergoing surgical treatment, patients were stratified according to whether surgical resection was performed or not. The scope of regional lymph node surgery was added as a confounder in the surgical resection secondary analysis. Patients with missing information regarding lymph node surgery were excluded from this analysis. Finally, the prognostic value of fibrosis was compared against other possible prognostic factors including pathological grade and tumor size.

A *p*-value below 0.05 was considered to be statistically significant. All statistical analysis was performed using the R Statistical Software (version 3.6.1; R Foundation for Statistical Computing, Vienna, Austria).

## CONCLUSIONS

Our results identify liver fibrosis as a strong prognostic factor in iCCA. Therefore, assessment of fibrosis level in iCCA patients upon diagnosis is of high importance, in order to improve risk stratification and establish treatment strategies.

## SUPPLEMENTARY MATERIALS



## Abbreviations

AJCC: American Joint Committee on Cancer
CCA: Cholangiocarcinoma
CCS: Cancer specific survival
CI: Confidence interval
dCCA: distal Cholangiocarcinoma
DCCPS: Division of Cancer Control and Population Sciences
HSC: Hepatic stellate cells
HR: Hazard ratio
iCCA: intrahepatic cholangiocarcinoma
ICD-O-3: International Classification of Diseases for Oncology, third edition
OS: Overall survival
PDT: Photodynamic therapy
PDGF: Platelet derived growth factor
pCCA: proximal Cholangiocarcinoma
RFA: Radio frequency ablation
SEER: Surveillance, Epidemiology, and End Results
TGF-β: Transforming growth factor beta
VEGF: Vascular endothelial growth factor
