# Supplementary material for: Effect of liver fibrosis on survival in patients with intrahepatic cholangiocarcinoma: a SEER population-based study
Source: Oncotarget. 2020 Nov 24;11(47):4438–47. doi: 10.18632/oncotarget.27820 (PMC7720776; doi:10.18632/oncotarget.27820)
Supplement: Supplementary file 1 [file oncotarget-11-4438-s001.pdf]

Effect of liver fibrosis on survival in patients with intrahepatic cholangiocarcinoma: a SEER population-based study

SUPPLEMENTARY MATERIALS

Supplementary Table 1: Comparison of patient characteristics between patients with a calculated fibrosis score and those without

	level	With fibrosis score	Without fibrosis score	*p*	SMD
n		512	3552		
Age at diagnosis (years) (mean (SD))		63.42 (11.17)	64.30 (12.51)	0.13	0.08
Gender (%)	Male	278 (54.3)	1762 (49.6)	0.05	0.09
	Female	234 (45.7)	1790 (50.4)		
Ethnicity (%)	White	390 (76.2)	2709 (76.3)	0.57	0.05
	Black	38 (7.4)	304 (8.6)		
	Other	84 (16.4)	539 (15.2)		
Year of diagnosis (mean (SD))		2011.37 (3.08)	2010.92 (3.34)	0	0.14
AFP (%)	Negative/normal	262 (73.0)	1215 (71.1)	0.51	0.04
	Positive/elevated	97 (27.0)	494 (28.9)		
Pathologic grade (%)	well_differentiated	36 (20.0)	157 (17.6)	0.52	0.06
	moderately_differentiated	144 (80.0)	734 (82.4)		
AJCC stage (%)	I	152 (29.7)	611 (17.2)	< 0.001	0.38
	II	47 (9.2)	249 (7.0)		
	III	148 (28.9)	956 (26.9)		
	IV	165 (32.2)	1736 (48.9)		
SEER spread summary (%)	Localized	193 (37.7)	777 (21.9)	< 0.001	0.36
	Regional - direct extension	53 (10.4)	413 (11.6)		
	Regional - lymph nodes	40 (7.8)	290 (8.2)		
	Regional - DEandLN	24 (4.7)	171 (4.8)		
	Distant	202 (39.5)	1901 (53.5)		
Surgery (%)	None	308 (60.2)	2788 (78.5)	< 0.001	0.41
	Liver_transplantation	10 (2.0)	18 (0.5)		
	Surgical_Resection	174 (34.0)	689 (19.4)		
	Tumor_destruction	20 (3.9)	57 (1.6)		
